# Antioxidant and Anti-Inflammatory Activity on LPS-Stimulated RAW 264.7 Macrophage Cells of White Mulberry (*Morus alba* L.) Leaf Extracts

**DOI:** 10.3390/molecules28114395

**Published:** 2023-05-28

**Authors:** Sureeporn Suriyaprom, Pitchayuth Srisai, Varachaya Intachaisri, Thida Kaewkod, Jeeraporn Pekkoh, Mickaël Desvaux, Yingmanee Tragoolpua

**Affiliations:** 1 Department of Biology, Faculty of Science, Chiang Mai University, Chiang Mai 50200, Thailand; sureeporn_suriyaprom@cmu.ac.th (S.S.); varachaya_in@cmu.ac.th (V.I.); thida.kaewkod@cmu.ac.th (T.K.); jeeraporn.p@cmu.ac.th (J.P.); 2 Graduate School, Chiang Mai University, Chiang Mai 50200, Thailand; pitchayuth_sri@cmu.ac.th; 3 INRAE, UCA, UMR0454 MEDIS, 63000 Clermont-Ferrand, France; mickael.desvaux@inrae.fr; 4 Natural Extracts and Innovative Products for Alternative Healthcare Research Group, Faculty of Science, Chiang Mai University, Chiang Mai 50200, Thailand

**Keywords:** antioxidation, anti-inflammation, oxyresveratrol, resveratrol, white mulberry

## Abstract

The white mulberry (*Morus alba* L.) is widely used as a medicinal plant in Asia. In this study, the bioactive compounds of ethanolic extracts of white mulberry leaves from the Sakon Nakhon and Buriram cultivars were evaluated. The ethanolic extracts of mulberry leaves from the Sakon Nakhon cultivar showed the highest total phenolic content of 49.68 mg GAE/g extract and antioxidant activities of 4.38 mg GAE/g extract, 4.53 mg TEAC/g extract, and 92.78 mg FeSO_4_/g extract using 2,2 diphenyl-1-picrylhydrazyl (DPPH), 2,20-azinobis-(3-ethylbenzothiazolin-6-sulfonic acid) (ABTS), and ferric reducing antioxidant power (FRAP) assays, respectively. The resveratrol and oxyresveratrol compounds in mulberry leaves were also investigated by high-performance liquid chromatography (HPLC). The mulberry leaf extracts from the Sakon Nakhon and Buriram cultivars showed oxyresveratrol contents of 1.20 ± 0.04 mg/g extract and 0.39 ± 0.02 mg/g extract, respectively, whereas resveratrol was not detected. It was also found that the potent anti-inflammatory properties of mulberry leaf extracts and its compounds, resveratrol and oxyresveratrol, suppressed the LPS-stimulated inflammatory responses in RAW 264.7 macrophage cells by significantly reducing nitric oxide production in a concentration-dependent manner. These compounds further inhibited interleukin-6 (IL-6) and tumor necrosis factor-α (TNF-α) production and suppressed the mRNA and protein expression of inducible nitric oxide synthase (iNOS) and cyclooxygenase-2 (COX-2) in LPS-stimulated RAW 264.7 macrophage cells. Therefore, it is established that mulberry leaf extract and its bioactive compounds contribute to its anti-inflammatory activity.

## 1. Introduction

Inflammation is a body response mechanism of the immune system that can be triggered by infectious agents, such as bacteria and viruses, as well as particles, toxic compounds, or damaged cells [1,2]. The regulation of inflammation is related to immune function and induced by inflammatory mediators that produce nitric oxide (NO) and cyclooxygenase (COX-2) [3]. Moreover, proinflammatory mediators including interleukin-6 (IL-6) and tumor necrosis factor-α (TNF-α) are generated by the activated macrophages, which promote the development of inflammation [4]. TNF-α, a very important pro-inflammatory cytokine, plays a pivotal role in the induction and persistence of inflammation and has numerous cellular effects [5,6]. Another cytokine which exerts an inflammatory stimulus is IL-6. These cytokines are responsible for the acute phase response and are known to contribute to tissue damage and multiple organ failure [7]. Lipopolysaccharide (LPS) and interferon-gamma stimulated murine macrophages lead to inducible nitric oxide synthase (*iNOS*) gene expression, which induces massive nitric oxide production from L-arginine and molecular oxygen as substrates and NADPH [8]. Inflammation can occur in many diseases, e.g., asthma, arthrophlogosis, and arthritis [4].

**Figure 1:**
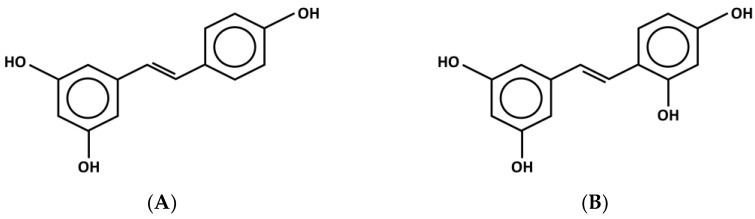
Chemical structure of (**A**) resveratrol (3,5,4′-trihydroxy-trans-stilbene) and (**B**) oxyresveratrol (2,3′,4,5′tetrahydroxy-trans-stilbene).

Over the last few years, there has been increasing attention on the anti-inflammatory effects of phytochemical compounds in plant extracts that are used in traditional medicine, since inflammation is recognized as playing an important role in several diseases. *Morus alba* L., or white mulberry, belongs to the genus *Morus* in the Moraceae family. Mulberry is a medicinal plant in Asia and is economically important to the silk industry as it serves as food for the silkworm (*Bombyx mori*) [9]. Mulberry leaves are highly nutritious, exhibit excellent antimicrobial, anti-diabetic, and anti-hypertensive activities, are known to lower blood sugar and cholesterol, and prevent acute and chronic diseases [10]. Their leaves possess several anti-inflammatory compounds, such as flavonoids and antioxidants as well as hydroxystilbenes, e.g., mulberroside A, resveratrol, kuwanon Y, kuwanon Z, and oxyresveratrol and its derivatives [11,12]. Resveratrol and its derivative, oxyresveratrol, are stilbenoids (Figure 1) [13]. These two similarly structured compounds are thought to have antioxidant, antiaging, antimutagenic [14], antidiabetic, and apoptotic properties, and are cancer and cardiovascular disease preventatives [15] which are linked by their important anti-inflammatory activities [16]. Resveratrol has an anti-inflammatory effect which decreases cytokine production, decreases proinflammatory mediator expression, alternates the expression of adhesion molecules, and inhibits neutrophil activity [17]. Resveratrol is involved in intracellular signaling pathways. This includes stimulating estrogen receptors and regulating the sirtuin 1 (SIRT1)/nuclear factor-kappa B (NF-*κ*B) [18,19] and the mitogen-activated protein kinases (MAPK)/hemeoxygenase-1 (HO-1) pathways [20,21]. Concerning oxyresveratrol, this compound demonstrates an inhibitory effect on prostaglandin E2 (PGE_2_) production and inhibits *iNOS* and *COX-2* gene expression through the suppression of NF-*κ*B binding activity [22].

The white mulberry in Thailand is primarily grown for its leaves, which are the preferred food of silkworms. Some people consume mulberry leaf tea as a healthy beverage that is a rich source of antioxidants. Mulberry leaves contain high levels of phenolics, flavonoids, and 1-deoxynojirimycin (DNJ) which possess beneficial effects in protecting against cardiometabolic risks, including obesity, hyperglycaemia, hypertension, hyperlipidaemia, inflammation, and oxidants, and have cardioprotective effects [10]. Mulberry trees are well adapted to their local climate conditions and have a variety of species such as Buriram 60, Yai-buriram, Sakon Nakhon, Nakhonratchasima 60, Khunpai, and Srisaket [23]. In this study, the bioactive compounds in ethanolic mulberry leaf extracts from the Sakon Nakhon and Buriram cultivars were estimated by measuring the total phenolic, flavonoid, and antioxidant activity. The resveratrol and oxyresveratrol contents of mulberry leaf extracts that provide the most effective anti-inflammatory activity were analyzed by high-performance liquid chromatography (HPLC). The anti-inflammatory activity of mulberry leaf extract as well as resveratrol and oxyresveratrol were further investigated, in particular, the regulation of iNOS and COX-2 mRNA and the protein expression in RAW 264.7 macrophage cells stimulated by lipopolysaccharide (LPS).

## 2. Results

### 2.1. Potential Bioactive Compounds and Antioxidant Activity of White Mulberry Leaf Extracts

The bioactive compounds in white mulberry leaf extracts were identified by evaluating the total phenolic, flavonoid, and antioxidant activity. The results are presented in Table 1. The total phenolic content of mulberry leaf extract from the Sakon Nakhon and Buriram cultivar was measured as 49.68 mg of gallic acid equivalent per gram of extract (mg GAE/g extract) and 27.27 mg GAE/g extract, respectively. Meanwhile, the total flavonoid content of mulberry leaf extract from the Sakon Nakhon and Buriram cultivar was measured as 0.90 mg of quercetin equivalent per gram of extract (mg QE/g extract) and 1.46 mg QE/g extract, respectively. The antioxidant activity of the extracts also confirmed the bioactivity of the phenolic and flavonoid content (Table 2). The DPPH radical scavenging activity and ABTS radical cation decolorization activity were expressed as IC_50_, which is the concentration of the extracts that inhibit the initial free radical by 50%. The lower IC_50_ value indicated a greater potential of the substances for scavenging DPPH and ABTS, implying a stronger antioxidant activity. The IC_50_ value of both the DPPH and ABTS activity was lower when taken from mulberry leaf extract from the Sakon Nakhon cultivar, showing 0.78 mg/mL by the DPPH assay and 4.89 mg/mL by the ABTS assay, indicating a higher antioxidant activity. Meanwhile, the IC_50_ value of mulberry leaf extract from the Buriram cultivar showed 1.67 mg/mL by the DPPH assay and 15.08 mg/mL by the ABTS assay. Moreover, the antioxidant activities were also compared to the standard gallic acid in the DPPH, Trolox in the ABTS, and ferrous sulphate (FeSO_4_) in the FRAP assays. Mulberry leaf extract from the Sakon Nakhon cultivar exhibited higher antioxidant activity than the Buriram cultivar, with an activity of 4.38 mg GAE/g extract, 4.53 milligrams of Trolox equivalent antioxidant capacity (TEAC) per gram of extract (mg TEAC/g extract), and 92.78 milligrams of ferrous sulfate per gram of extract (mg FeSO_4_/g extract) (Table 2). This finding indicated that the highest antioxidant activity of mulberry leaf extract from the Sakon Nakhon cultivar was associated with the highest phenolic content.

**Figure 2:**
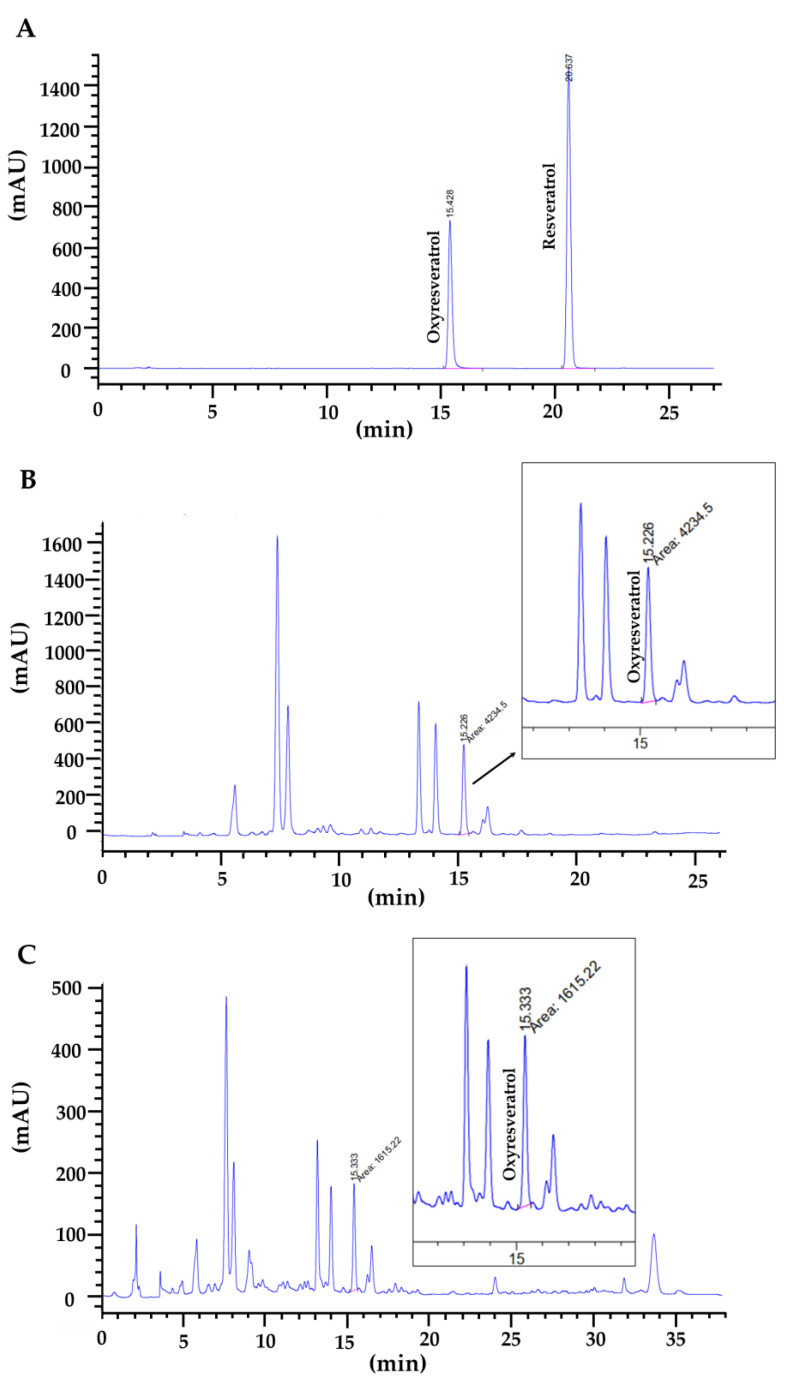
HPLC chromatogram of (**A**) standard mixture of oxyresveratrol and resveratrol; (**B**) white mulberry leaf extract from Sakon Nakhon cultivar; and (**C**) Buriram cultivar.

Although numerous studies have shown that the main bioactive components of mulberries are phenolics and flavonoids [24], these plants are also rich in resveratrol and oxyresveratrol, which demonstrate antioxidant, antibacterial, and anti-inflammatory properties [25]. In this study, the levels of oxyresveratrol and resveratrol in mulberry leaf extract were quantified by an HPLC assay. The oxyresveratrol found in mulberry leaf extract from the Sakon Nakhon cultivar was measured at 1.20 ± 0.04 mg/g extract and 0.39 ± 0.02 mg/g extract from the Buriram cultivar, whereas resveratrol was not detected in this study (Figure 2).

### 2.2. Effects of White Mulberry Leaf Extract, Resveratrol, and Oxyresveratrol on Cell Viability

**Figure 3:**
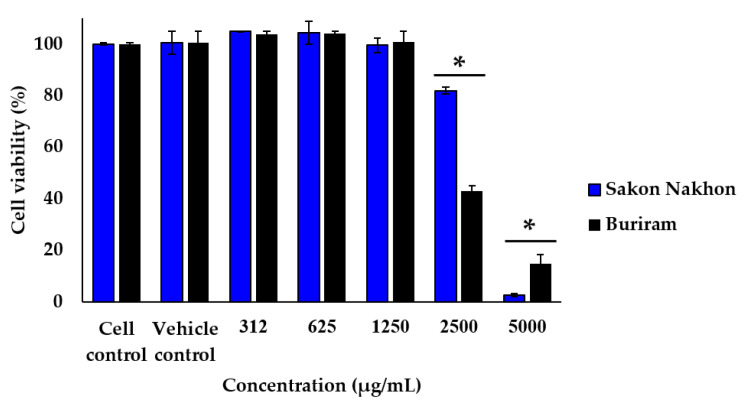
Effect of white mulberry leaf extract on the viability of RAW 264.7 cells. Cells were treated with the indicated concentration (312, 625, 1250, 2500, and 5000 µg/mL) of mulberry leaf extract for 24 h, and cell viability was assessed by MTT assay. The results are expressed as the percentage of surviving cells over control cells. Each value indicates the mean ± SD (*n* = 3). * indicates significant difference (*p* < 0.05).

**Figure 4:**
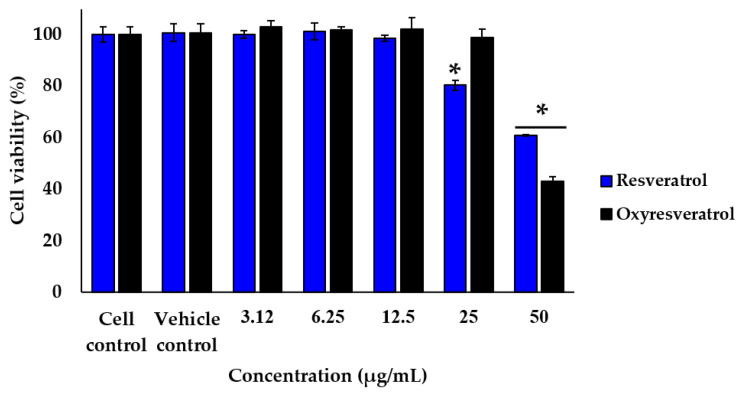
Effect of resveratrol and oxyresveratrol on the viability of RAW 264.7 cells. Cells were treated with the indicated concentration (3.12, 6.25, 12.5, 25, and 50 µg/mL) of resveratrol and oxyresveratrol for 24 h, and cell viability was assessed by MTT assay. The results are expressed as the percentage of surviving cells over control cells. Each value indicates the mean ± SD (*n* = 3). * indicates significant difference (*p* < 0.05).

The effect of white mulberry leaf extracts on cell viability was determined after incubating cells with ethanolic mulberry leaf extract at concentrations of 312, 625, 1250, 2500 and 5000 µg/mL for 24 h. The results of the MTT assay indicated that mulberry leaf extract at a concentration of up to 1250 µg/mL had no significant toxicity effects on the viability of RAW 264.7 cells. The effect of resveratrol and oxyresveratrol on cell viability was determined after incubating cells at concentrations of 3.12, 6.25, 12.5, 25 and 50 µg/mL. The results showed that resveratrol and oxyresveratrol were also nontoxic on RAW 264.7 cells at concentrations of up to 12.5 and 25 µg/mL, respectively. Therefore, these concentrations of mulberry leaf extract as well as resveratrol and oxyresveratrol were chosen for further experiments (Figure 3 and Figure 4).

### 2.3. Effects of White Mulberry Leaf Extract, Resveratrol, and Oxyresveratrol on LPS-Stimulated Nitric Oxide Production

**Figure 5:**
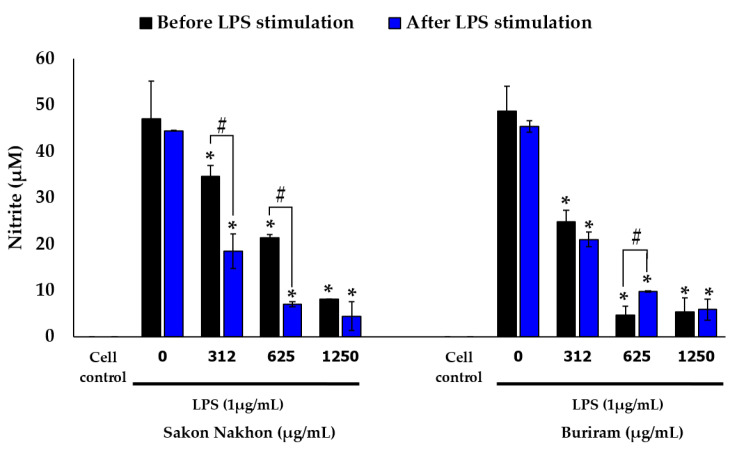
Effects of white mulberry leaf extract on nitric oxide production in LPS-stimulated RAW 264.7 cells. The amount of nitric oxide produced was then determined using Griess assay. The results are expressed as mean ± SD (*n* = 3). * indicates significant difference (*p* < 0.05) compared with the cells treated with LPS alone (LPS group). # indicates significant difference (*p* < 0.05) before and after LPS stimulation.

**Figure 6:**
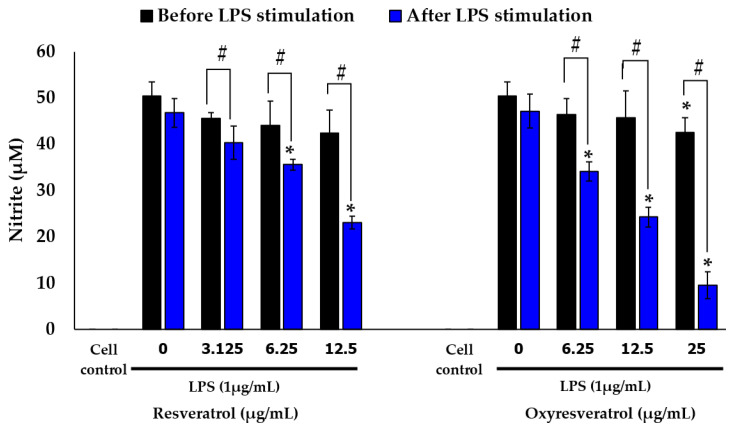
Effects of resveratrol and oxyresveratrol on nitric oxide production in LPS-stimulated RAW 264.7 cells. The amount of nitric oxide produced was then determined using Griess assay. The results are expressed as mean ± SD (*n* = 3). * indicates significant difference (*p* < 0.05) compared with the cells treated with LPS alone (LPS group). # indicates significant difference (*p* < 0.05) before and after LPS stimulation.

The effects of mulberry leaf extract, resveratrol, and oxyresveratrol on LPS-stimulated nitric oxide production on RAW 264.7 cells are shown in Figure 5 and Figure 6. Cells were treated with the mulberry leaf extract, resveratrol, and oxyresveratrol before the LPS stimulation for the prevention of inflammation, and the cells were also treated with the mulberry leaf extract, resveratrol, and oxyresveratrol after the LPS stimulation to clarify the treatment of inflammation. Sodium nitrite was used as a standard to determine the concentration of nitrite, and the amount of nitrite present in the culture supernatants was defined as an indirect measurement of nitric oxide production. The results indicated that mulberry leaf extracts from both the Sakon Nakhon and Buriram cultivars significantly reduced nitric oxide production in a concentration-dependent manner, and the nitrite levels were found between 4.43 and 48.77 µM (Figure 5). Mulberry leaf extract from the Sakon Nakhon cultivar significantly decreased the nitrite levels 1.36-, 2.21-, and 5.80-fold at concentrations of 312, 625, and 1250 µg/mL when treated before stimulation with LPS. However, when the mulberry leaf extract from the Sakon Nakhon cultivar was treated after stimulation with LPS at concentrations of 312, 625, and 1250 µg/mL, the nitrite levels significantly decreased 2.40-, 6.37-, and 10.03-fold, respectively. Furthermore, mulberry leaf extract from the Buriram cultivar at concentrations of 312, 625, and 1250 µg/mL also showed significantly reduced nitrite levels; they decreased 1.96-, 10.33-, and 9.00-fold when treated before stimulation with LPS and 2.16-, 4.61-, and 7.68-fold after stimulation with LPS, respectively, compared to the cells treated with LPS alone.

Similarly, the results showed that resveratrol and oxyresveratrol were able to inhibit nitric oxide production in a concentration-dependent manner and the nitrite levels were found to be between 9.54 and 50.42 µM (Figure 6). Additionally, treating the compounds after stimulation with LPS could inhibit nitric oxide more effectively than treating the compounds before stimulation with LPS. Resveratrol at concentrations of 6.25 and 12.5 µg/mL significantly reduced the nitrite levels 1.31- and 2.02-fold when treated after stimulation with LPS. Oxyresveratrol at concentrations of 6.25, 12.5, and 25 µg/mL significantly reduced the nitrite levels when treated after stimulation with LPS 1.38-, 1.94-, and 4.94-fold, respectively. For the untreated RAW 264.7 cells (the cell control), the concentration of nitrite could not be detected.

### 2.4. Effects of White Mulberry Leaf Extract, Resveratrol, and Oxyresveratrol on the mRNA Expression of iNOS and COX-2 in LPS-Stimulated RAW 264.7 Cells

**Figure 7:**
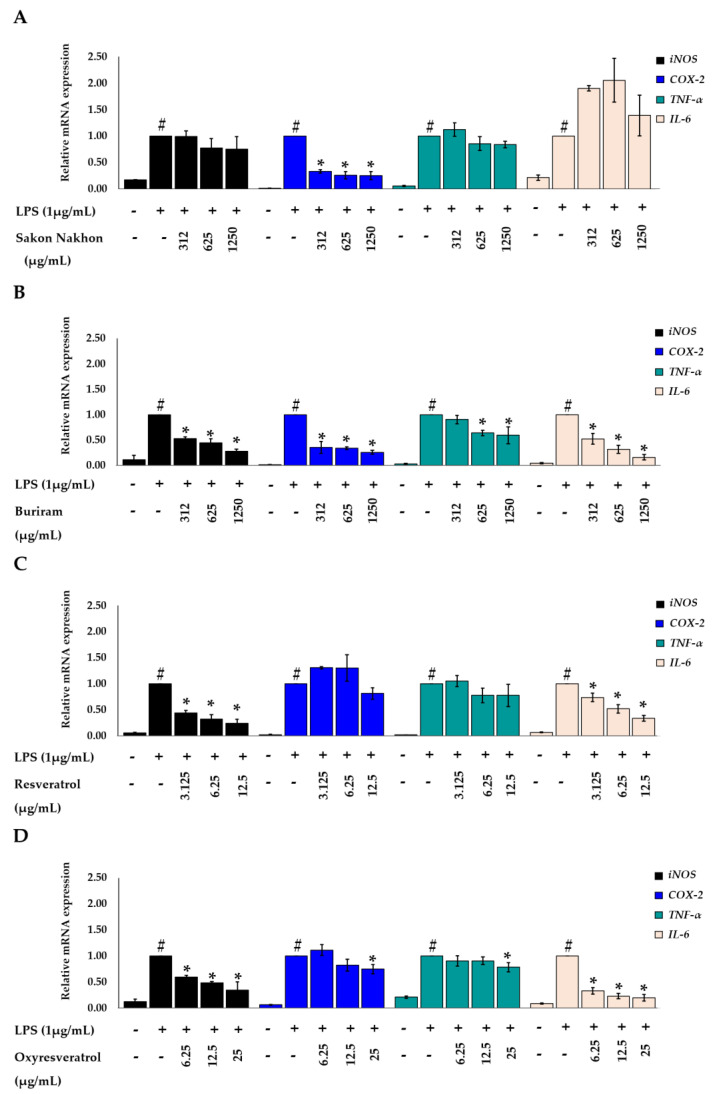
Effects of (**A**) mulberry leaf extract from Sakon Nakhon cultivar, (**B**) mulberry leaf extract from Buriram cultivar, (**C**) resveratrol, and (**D**) oxyresveratrol on the relative mRNA expression levels of *iNOS*, *COX-2*, *TNF-α*, and *IL-6* in LPS-stimulated RAW 264.7 cells. Cells were stimulated with LPS (1 µg/mL) for 30 min and then treated with the extract for 3 h. The mRNA levels were measured by qRT-PCR. Data are expressed as mean ± SD (*n*  = 3). # indicates significant difference (*p* < 0.05) compared with the cells untreated with LPS (cell control). * indicates significant difference (*p* < 0.05) compared with the cells treated with LPS alone (LPS group).

The effects of mulberry leaf extract, resveratrol, and oxyresveratrol on the mRNA expression of *iNOS* and *COX-2* in LPS-stimulated RAW 264.7 cells were evaluated using real-time quantitative PCR. The results revealed that mulberry leaf extract inhibited the mRNA expression of *iNOS* and *COX*-2 in a concentration-dependent manner (Figure 7). In the cells treated with mulberry leaf extracts from both the Sakon Nakhon and Buriram cultivars at a concentration of 312–1250 µg/mL, the expression of the *COX-2* level decreased by more than 50% compared with that in cells treated with LPS alone (*p* < 0.05). Moreover, the highest concentration of mulberry leaf extract at 1250 µg/mL had the greatest inhibitory effect on the mRNA expression of *iNOS* and *COX-2*. Regarding resveratrol and oxyresveratrol, these compounds were able to inhibit the mRNA expression of *iNOS* in a concentration-dependent manner. Resveratrol at a concentration of 6.25–12.5 µg/mL and oxyresveratrol at a concentration of 12.5–25 µg/mL could suppress the mRNA expression of *iNOS* by more than 50% and inhibited *COX-2* when treated with resveratrol and oxyresveratrol at concentrations of 12.5 and 25 µg/mL, respectively.

### 2.5. Effects of White Mulberry Leaf Extract, Resveratrol, and Oxyresveratrol on the mRNA Expression of TNF-α and IL-6 in LPS-Stimulated RAW 264.7 Cells

The inhibitory effects of mulberry leaf extract, resveratrol, and oxyresveratrol on the production of pro-inflammatory cytokines were further investigated with consideration of the transcription of *TNF-α* and *IL-6* using real-time quantitative PCR. As shown in Figure 7, the mulberry leaf extract, resveratrol, and oxyresveratrol downregulated the expression levels of *TNF-α* and inhibited the expression levels of *IL-6* in a concentration-dependent manner. The mulberry leaf extract from the Buriram cultivar at a concentration of 625–1250 µg/mL, the resveratrol at a concentration of 12.5 µg/mL, and the oxyresveratrol at a concentration of 6.25–25 µg/mL could suppress the mRNA expression of *IL-6* by more than 50%, compared to the cells treated with LPS alone (*p* < 0.05). However, the mulberry leaf extract from the Sakon Nakhon cultivar did not downregulate the mRNA expression levels of *IL-6* (*p* < 0.05) in the same conditions. The expression of the housekeeping gene *β-actin* was not induced by the treatment.

### 2.6. Effects of White Mulberry Leaf Extract, Resveratrol, and Oxyresveratrol on the Protein Expression of iNOS and COX-2 in LPS-Stimulated RAW 264.7 Cells

**Figure 8:**
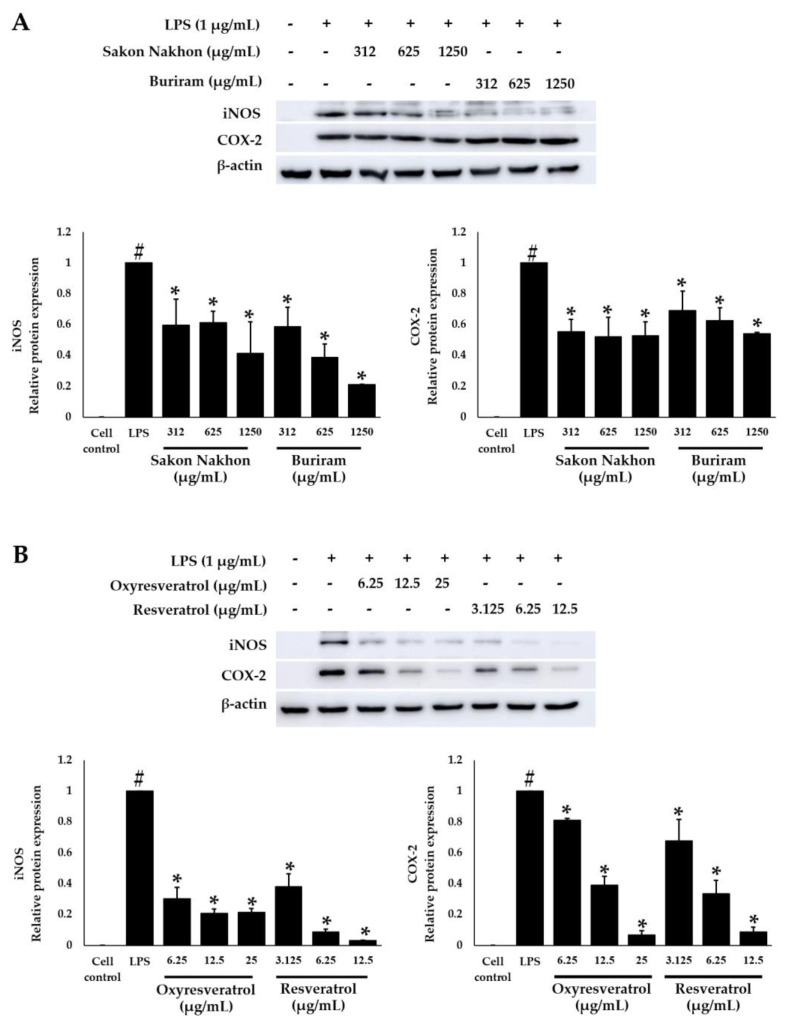
Effects of (**A**) white mulberry leaf extracts from the Sakon Nakhon and Buriram cultivars, and (**B**) oxyresveratrol and resveratrol on the protein expression of COX-2 and iNOS in LPS-stimulated RAW 264.7 cells. Cells were stimulated with LPS (1 µg/mL) for 30 min and then treated with the extract for 12 h. Cell lysates (30 μg/mL) were separated by SDS-PAGE, transferred to a PVDF membrane, and detected with anti-COX-2, anti-iNOS, and β-actin antibodies. β-actin was used as an internal control. Data are expressed as mean ± SD (*n*  = 3). # indicates significant difference (*p* < 0.05) compared with the cells untreated with LPS (cell control). * indicates significant difference (*p* < 0.05) compared with the cells treated with LPS alone (LPS group).

The effects of mulberry leaf extract, resveratrol, and oxyresveratrol on the protein expression of iNOS and COX-2 in LPS-stimulated RAW 264.7 cells were determined by a Western blot analysis. The mulberry leaf extracts from both the Sakon Nakhon and Buriram cultivars reduced the protein expression of iNOS and COX-2 (*p* < 0.05) (Figure 8A). The mulberry leaf extract from the Sakon Nakhon cultivar at a concentration of 1250 µg/mL and the Buriram cultivar at a concentration of 625–1250 µg/mL could suppress the protein expression of iNOS by more than 50%. Regarding the standard compounds, oxyresveratrol and resveratrol inhibited iNOS and COX-2 proteins in a concentration-dependent manner (*p* < 0.05) (Figure 8B). The resveratrol at a concentration of 3.125–12.5 µg/mL and the oxyresveratrol at a concentration of 6.25–25 µg/mL reduced the protein expression of iNOS by more than 50% (*p* < 0.05) and also downregulated the COX-2 expression by more than 50% at concentrations of 6.25–12.5 and 12.5–25 µg/mL of resveratrol and oxyresveratrol, respectively. Furthermore, the inhibition of the protein expression of iNOS and COX-2 by the mulberry leaf extract as well as oxyresveratrol and resveratrol correlated well with the *iNOS* and *COX-2* mRNA expression.

## 3. Discussion

Numerous studies have confirmed that the phenolics and flavonoids found throughout medicinal plants [24] are major bioactive components and have antioxidant and anti-inflammatory properties [25]. In this study, the total phenolic and flavonoid content in white mulberry leaf extract was found in different proportions. Mulberry leaf extract was found to be plentiful in phenolic compounds, higher than previously reported, and the phenolic content was also found to be higher than that in mulberry leaves cultivated in various region of Korea [26]. High phenolic content is the main contributor to antioxidant activity in mulberry leaves. The antioxidant activity was detected by complementary assays, including DPPH, ABTS, and FRAP assays. As previously revealed, the antioxidant activity was higher in mulberry leaf extracts from the Sakon Nakhon cultivar. These extracts are likely to contribute to the anti-inflammatory activity in this study. Moreover, resveratrol and oxyresveratrol have been reported to have anti-inflammatory activity. The levels of resveratrol and oxyresveratrol in mulberry leaf extract were analyzed by an HPLC assay, and only oxyresveratrol was detected in this study. Many parts of the mulberry were found to contain higher levels of oxyresveratrol than resveratrol. These results were consistent with reports of higher oxyresveratrol levels in mulberry extract from China, whereas the resveratrol levels were either very low or not detected [12].

Phenolic and flavonoid compounds are confirmed to exhibit antioxidant [27], anti-inflammatory [28], and antibacterial activity [29]. Additionally, the main bioactive compounds in mulberry leaves, e.g., mulberroside A, resveratrol, and oxyresveratrol [12], possess antioxidant and anti-inflammatory properties [22,30]. Inflammation, especially chronic inflammation, is the greatest threat to human health and the leading cause of death in the world. Many people suffer from chronic inflammatory diseases, such as stroke, heart disease, obesity, diabetes, chronic respiratory disease, and cancer [31]. Anti-inflammatory drugs, namely non-steroidal anti-inflammatory drugs (NSAIDs), have been developed to restrain the cyclooxygenases (COXs) to inhibit prostaglandins [32]. However, many critical adverse effects on the gastric mucosa, cardiovascular system, renal system, hematologic system, and hepatic system occur during COX inhibition [33], therefore making it crucial to search for new alternatives to treat inflammation. Phytochemical compounds in plant extracts are increasing gaining attention as inflammatory treatments based on their broad spectrum of biological activity, as well as their safety and ability to reduce the risk of serious complications in long-term usage. In this study, white mulberry leaf extract and its bioactive compounds, resveratrol and oxyresveratrol, were further investigated for anti-inflammatory activity, revealing its efficacy to be developed as an alternative treatment for reducing inflammation.

Lipopolysaccharide (LPS) was required for the full activation of RAW 264.7 macrophage cells [34]. LPS can induce high levels of iNOS expression and increase nitric oxide (NO) production, which is the most important inflammatory mediator involved in the inflammatory process and is needed to maintain inflammatory responses. In particular, high levels of nitric oxide are characterized as cytotoxic molecules in inflammation and endotoxemia [35]. The production of nitric oxide is unstable and rapidly oxidizes to nitrite (NO_2_^−^). Therefore, it is defined by assessing the level of nitrite, commonly used as a surrogate marker of nitric oxide generation [36]. In this study, RAW 264.7 macrophage cells were treated with extracts for the protection of inflammation before LPS stimulation, and the cells were treated with extracts for the treatment of inflammation after LPS stimulation. After stimulation with LPS, the cells produced a group of enzymes called nitric oxide synthases. These enzymes convert arginine into citrulline, producing nitric oxide in the process [36]. Therefore, the treatment with the extract can inhibit the process of nitric oxide generation. The treatment extracts after being stimulated with LPS in RAW 264.7 macrophages had a greater ability to inhibit nitric oxide production than the treatment extracts before stimulation with LPS.

Moreover, the inhibitory effect on nitric oxide production in white mulberry leaf extracts from both the Sakon Nakhon and Buriram cultivars was higher than resveratrol and oxyresveratrol, since the extracts contain phenolic and flavonoid polyphenols. Apart from these polyphenols, oxyresveratrol possibly accounts for the high anti-inflammatory properties of mulberry leaf extract. White mulberry leaf extract, resveratrol, and oxyresveratrol were also found to suppress the *iNOS* mRNA expression level. These findings showed that nitric oxide inhibition may be due to the inhibition of *iNOS* during LPS-stimulation on macrophages [37]. The oxidoreductase enzyme, cyclooxygenase (COX), is also known as prostaglandin endoperoxide synthase, which can catalyze the conversion of arachidonate to prostanoids, such as prostaglandins (PGs), prostacylins, and thromboxane [38]. Therefore, it mediates the inflammatory response, and it is the target of NSAIDs [39]. In this study, our results exhibited that white mulberry leaf extracts from both the Sakon Nakhon and Buriram cultivars downregulated the expression level of iNOS and COX-2 in both mRNA and protein. Consequently, the transcription and translation levels of iNOS- and COX-2-derived inflammatory mediators in LPS-stimulated RAW 264.7 macrophages can be suppressed by mulberry leaf extract. On the other hand, the bioactive compounds, resveratrol and oxyresveratrol, target only *iNOS* and have no effect on *COX-2* mRNA expression. However, in a concentration-dependent manner, resveratrol and oxyresveratrol significantly suppressed the protein expression levels of both iNOS and COX-2. Thus, it is considered that resveratrol and oxyresveratrol might influence the process of iNOS and COX-2 translation from gene to protein. Additionally, these active compounds might inhibit the formation of prostaglandin E2 (PGE2) induced by LPS by reducing the expression level of the *COX-2* gene [40].

**Figure 9:**
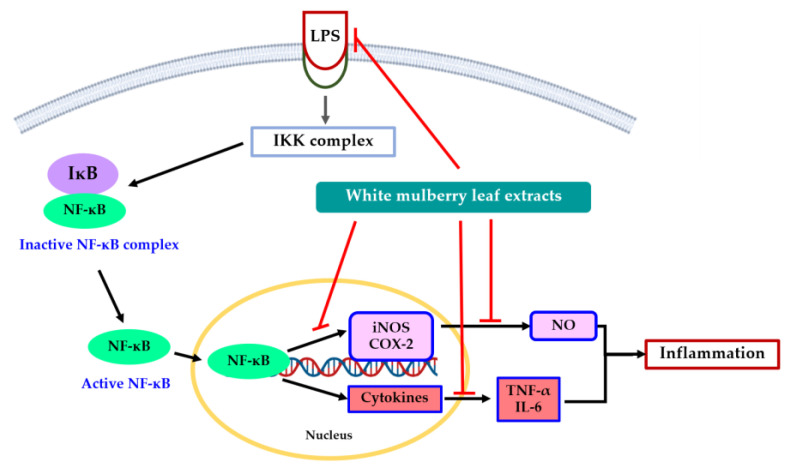
Proposed mechanism of action by which white mulberry leaf extract inhibits inflammation on LPS-stimulated RAW 264.7 macrophage cells.

The inhibition of pro-inflammatory cytokines by mulberry leaf extract was further examined for anti-inflammatory activity as well as its bioactive substances. The inflammatory response can be orchestrated by pro-inflammatory cytokines, such as TNF-α and IL-6, and it can be mediated through NF-κB transcriptional factor [41]. Nitric oxide production can induce TNF-α production in response to LPS stimulation, which can activate a cytokine cascade of inflammatory responses and stimulate IL-1β and IL-6 [42]. The critical pro-inflammatory cytokines, e.g., TNF-α and IL-6, that encourage the progression of inflammation were detected in this research. LPS stimulation elevated the production of all these inflammatory cytokines. The increase in these inflammatory cytokines was then considerably suppressed by the mulberry leaf extract from the Buriram cultivar, along with oxyresveratrol. Furthermore, the mulberry leaf extract from the Sakon Nakhon cultivar downregulated the level of *TNF-α*, but did not affect the expression of *IL-6*. A prior study found that NF-κB, a crucial transcriptional regulator, regulates the production of pro-inflammatory cytokines, iNOS and COX-2, at the transcriptional level [37], and is important for immunity, stress response, and inflammation. NF-κB dimers are rendered inactive in the cytoplasm through binding to the kappa B inhibiting protein (IκB). IκB activation occurs when stimulated by IL-1β, TNF-α, and LPS. The free form of NF-κB then translocates to the nucleus and performs as a transcription factor. NF-κB dimers engage with the targeted DNA regions in the nucleus to cause the transcription of gene-encoding inflammatory proteins, including iNOS and COX-2, resulting in the production of nitric oxide (NO) and prostaglandins (PGs) [43]. This finding indicate that mulberry leaf extract decreases the expression levels of iNOS, COX-2, and cytokine production. Therefore, the downregulation of iNOS, COX-2, and cytokines, e.g., TNF-α and IL-6, could be caused by the suppression of NF-κB activation (Figure 9) [44].

Among the phytochemical compounds in mulberry leaf extracts, resveratrol and oxyresveratrol have sparked a lot of curiosity based on their abilities for antioxidant, antibacterial, and anti-inflammatory activity. Our research indicated that these bioactive compounds appeared to decrease the expression of iNOS and COX-2 proteins better than crude mulberry leaf extract. This might be due to the amount of resveratrol and oxyresveratrol in the test being greater than the compound content that found in the mulberry leaf extract. Although, in this study, only oxyresveratrol was detected in the mulberry leaf extract, the inhibitory ability of these compounds has also been supported in other studies [30]. Resveratrol has been extensively studied for the treatment of respiratory, metabolic, and cardiovascular diseases, as well as cancer and neurodegenerative diseases [16]. It was found that resveratrol interacts with several molecular targets, most of which are related to various immune-mediated diseases, such as inflammation and immunity. Resveratrol plays a direct role on both innate and adaptive immunity [45]. It can inhibit COX-2 directly by reducing COX-2 enzyme activity, which is dose-dependent on PGE2 production, and inhibits COX-2 indirectly via ERK-1 and c-Jun on the MAPK pathway [46]. It also influences the NF-κB pathway, as it prevents TNF-α-induced NF-κB activation in a dose- and time-dependent manner. Considering the NF-κB inhibitory mechanism of resveratrol, it appears that resveratrol greatly reduced LPS-induced nitric oxide formation as well as the mRNA and protein levels of iNOS in RAW 264.7 cells. This may be due to lowered phosphorylation and the degradation of IκB-α, which impairs the subsequent nuclear translocation of the NF-κB subunit and DNA binding [47,48]. In addition, resveratrol inhibits COX-2 expression, which has consequences for prostaglandin synthesis [49]. It can inhibit TNF-α and IL-6, thereby reducing inflammation-inducing NF-κB levels [50]. Regarding oxyresveratrol, similar biological activity was reported. Oxyresveratrol exerts anti-inflammatory properties by inhibiting both the mRNA and protein levels of IL-6 and IL-8 [51] and also inhibits NF-κB activation, iNOS and nitric oxide production, and PGE2 synthesis [22]. Oxyresveratrol also suppresses IκB-α phosphorylation, JNK and p38 phosphorylation, and NF-κB nuclear translocation, thereby blocking the NF-κB and MAPK signaling pathways [30].

Apart from resveratrol and oxyresveratrol, numerous natural antioxidant substances have been found to inhibit NF-κB-dependent cytokines as well as iNOS and COX-2 levels. The suppressive influence on the generation of inflammatory mediators is linked to their antioxidant activity [52]. Among the biologically active compounds in mulberry leaves, compounds showing antioxidant activity, e.g., phenolic acids and flavonoids, have received special attention for their anti-inflammatory properties [53]. In this study, phenolic compounds performed similarly to NSAIDs, and in addition to COX, they also reduced the activity and gene expression of other pro-inflammatory mediators. Additionally, some phenolic and flavonoid compounds can attenuate transcription factors of iNOS and COX-2 through the inhibition of NF-κB activity in the inflammatory pathway [54]. Hence, bioactive compounds in white mulberry leaf extract, e.g., phenolic and flavonoid compounds as well as oxyresveratrol, and antioxidant activity contribute to anti-inflammation in this study.

## 4. Materials and Methods

### 4.1. Reagents and Chemicals

Folin-Ciocalteu reagent, TPTZ (2,4,6-tri(2-pyridyl)-s-triazine), and quercetin dehydrate were purchased from Merck (Billerica, MA, USA). 2,2-diphenyl-1-picrylhydrazil (DPPH), 2,2′-azinobis-(3-ethylbenzothiazolin-6 sulfonic acid) (ABTS), 6-hydroxy-2,5,7,8-tetramethyl-chroman-2-carboxylic acid (Trolox), gallic acid monohydrate, resveratrol, oxyresveratrol, and lipopolysaccharide (LPS) from *Escherichia coli* O111:B4 were obtained from Sigma-Aldrich (St. Louis, MO, USA). RAW 264.7 cell culture media and Dulbecco’s Modified Eagle’s Medium (DMEM) were purchased from Gibco (Grand Island, NY, USA). 3-(4,5-dimethylthizaol-2-yl)-2,5-diphenyl tetrazolium bromide (MTT) was obtained from Bio Basic (Toronto, ON, Canada). Primary antibodies, anti-beta-actin monoclonal antibody, anti-iNOS/NOS II polyclonal antibody, and anti-COX-2 polyclonal antibody, as well as the peroxidase-conjugated secondary antibody were purchased from EMD Millipore corporation (Temecula, CA, USA).

### 4.2. Materials

White mulberry leaves (*M. alba* L.) from Sakon Nakhon and Buriram cultivars were collected during May–June in Chiang Mai province (northern Thailand: 19°00′ N, 99°00′ E) under the supervision of the Queen Sirikit Department of Sericulture. The leaves were dried in an oven at 60 °C for 48–72 h and blended into powder with a blender. The samples were kept in airtight plastic bags for around 1–2 days prior to extraction.

RAW 264.7 macrophage cell line originated from mice (*Mus musculus*) and was purchased from ATCC with the accession number ATCC-TIB-71.

### 4.3. Preparation of Extracts

Mulberry leaf powder was extracted with 95% ethanol in a ratio of 1 to 10 (*w*/*v*) and was shaken at 150 rpm (IKA, Staufen, Germany) for 72 h at room temperature. The extracts were then filtered through a Whatman No.1 filter paper and vaporized by a rotary evaporator (Heidolph, Schwabach, Germany) under a vacuum at 45 °C. Afterward, the extracts were freeze-dried by lyophilization (LABCONCO, Kansas City, MO, USA) and dissolved in dimethyl sulfoxide (DMSO) at a concentration of 500 mg/mL until they were used for experimentation. 

### 4.4. Bioactive Compounds in White Mulberry Leaf Extract

#### 4.4.1. Determination of Total Phenolic Content

Total phenolic content was investigated by the Folin–Ciocalteu method [55]. Briefly, mulberry leaf extracts (250 µL) were mixed with 125 µL of 50% Folin–Ciocalteu reagent, followed by the addition of distilled water (1.25 mL), 95% ethanol (250 µL), and 5% *w*/*v* sodium carbonate solution (250 µL). After incubation at room temperature for 1 h, the absorbance was measured at 725 nm using a spectrophotometer (Thermo Scientific, Waltham, MA, USA). The total phenolic content was expressed as milligrams of gallic acid equivalents (GAE) per 1 g of extract (mg GAE/g extract).

#### 4.4.2. Determination of Total Flavonoid Content

Total flavonoid content was determined by the aluminum chloride colorimetric method [56]. Briefly, mulberry leaf extracts (500 µL) were mixed with 100 µL of 10% aluminum chloride, followed by the addition of methanol (1.5 mL), 1 M potassium acetate (100 µL), and distilled water (2.8 mL). After incubating the mixture for 30 min at room temperature, the absorbance was measured at 415 nm using a spectrophotometer (Thermo Scientific, Waltham, MA, USA). Total flavonoid content was calculated as milligrams of quercetin equivalent (QE) per 1 g of extract (mg QE/g extract).

#### 4.4.3. DPPH Radical Scavenging Assay

The radical scavenging activity was evaluated using 2,2-diphenyl-1-picrylhydrazil (DPPH) assay [30] with minor modifications. Mulberry leaf extracts (500 µL) were mixed with 1.5 mL of 0.1 mM DPPH solution and incubated for 20 min in the dark at room temperature. The absorbance was measured at 517 nm using a spectrophotometer (Thermo Scientific, Waltham, MA, USA). The radical scavenging activity of mulberry leaf extracts is presented as IC_50_. Gallic acid was used as a standard and the antioxidant activity is presented as milligrams of gallic acid equivalent per 1 g of extract (mg GAE/g extract).

#### 4.4.4. ABTS Radical Cation Decolorization Assay

The radical cation decolorization activity was determined using 2,2′-azinobis-(3-ethylbenzothiazolin-6-sulfonic acid) (ABTS.^+^) assay [57] with some modifications. Mulberry leaf extracts (5 µL) were combined with 195 µL of ABTS.^+^ solution containing an absorbance of 0.700 ± 0.020 at 734 nm. After incubation for 10 min, the absorbance was measured at 734 nm using a microplate reader (DYNEX Technologies, Chantilly, VA, USA). The radical cation decolorization activity of the mulberry leaf extracts is presented as IC_50_. Trolox was used as a standard, and the antioxidant activity is presented as milligrams of Trolox equivalent antioxidant capacity (TEAC) per 1 g of extract (mg TEAC/g extract).

#### 4.4.5. Ferric Reducing Antioxidant Power (FRAP) Assay

The ferric reducing antioxidant power was estimated using reductants in a redox-linked colorimetric method [58]. The mulberry leaf extracts (500 µL) were mixed with 1.5 mL of FRAP reagent (300 mM acetate buffer pH 3.6, 20 mM ferric chloride solution, 10 mM TPTZ (2,4,6-tri(2-pyridyl)-s-triazine) in 40 mM hydrochloric acid, and distilled water). After incubation for 15 min in the dark at room temperature, the absorbance was measured at 593 nm using a spectrophotometer (Thermo Scientific, Waltham, MA, USA). Ferrous sulfate was used as a standard, and the antioxidant activity is presented as milligrams of ferrous sulfate (FeSO_4_) per 1 g of extract (mg FeSO_4_/g extract).

#### 4.4.6. Analysis of Resveratrol and Oxyresveratrol in Mulberry Leaf Extracts by HPLC

The resveratrol and oxyresveratrol found in mulberry leaf extracts were determined using high-performance liquid chromatography (HPLC) with some modifications [12]. These compounds were detected by gradient HPLC systems consisting of a conventional C18 column. Mulberry leaf extracts were filtered through a 0.22 µm sterile microfilter, and the filtrate (20 μL) was injected into the HPLC system (Agilent technologies 1200 series, Santa Clara, CA, USA). All analyses were carried out with a C18 column (4.6 × 150 mm, 5 µm, GL Sciences, Tokyo, Japan) at a temperature of 40 °C. Ultraviolet absorption was detected at 320 nm, and the HPLC system operated at a flow rate of 1.0 mL/min. Eluent A (1.0% aqueous formic acid, *v*/*v*) and B (acetonitrile) were used with the gradient program set as follows: 0–30 min, linear change from A-B (95:5, *v*/*v*) to A-B (70:30, *v*/*v*). The peak area of resveratrol and oxyresveratrol was calculated by the Agilent ChemStation level-5 program, and the content of each compound in the mulberry leaf extracts was calculated from the standard graph within a range of 7.8 to 125 µM (y = 139.744 x + 247.28, R^2^ = 0.9997) of resveratrol and a range of 7.8 to 125 µM (y = 70.541 x + 154.64, R^2^ = 0.9995) of oxyresveratrol.

### 4.5. Anti-Inflammatory Activity

#### 4.5.1. Cell Culture

RAW 264.7 macrophage cells were cultured in Dulbecco’s Modified Eagle’s Medium (DMEM) supplemented with 10% heat-inactivated fetal bovine serum (FBS) and 1% penicillin-streptomycin (100 U/mL penicillin and 100 µg/mL streptomycin) and maintained at 37 °C in a 5% CO_2_ incubator.

#### 4.5.2. Cell Viability Assay

Cell viability was determined by 3-(4,5-dimethylthiazol-2-yl)-2,5-diphenyl tetrazolium bromide (MTT) assay. Briefly, the cells were grown in 96-well plates at a density of 2 × 10^5^ cells/well and incubated at 37 °C in a 5% CO_2_ incubator for 24 h. The media were removed and the cells were then treated with 100 µL of the control medium or the medium supplemented with various concentrations of mulberry leaf extract (312–5000 µg/mL), resveratrol (3.12–50 µg/mL), or oxyresveratrol (3.12–50 µg/mL) and incubated at 37° for 24 h. MTT solution (2 mg/mL in phosphate buffer saline) was added to each well and the plates were incubated at 37 °C for 4 h.

Next, the medium from the wells was removed, and 200 µL of DMSO was added to the wells to solubilize the formed formazan crystals. The absorbance was measured on a microplate reader (DYNEX Technologies, Chantilly, VA, USA) using a test wavelength of 540 nm and a reference wavelength of 630 nm [3]. Non-toxic concentrations of mulberry leaf extract, resveratrol and oxyresveratrol that had no significant effect on cell viability of RAW 264.7 compared with the cell control group (more than 90% viability) were used to study anti-inflammatory activity.

#### 4.5.3. Nitrite Determination

*Treatment before stimulation with LPS*

The cells were plated at a density of 2 × 10^5^ cells/well in 96-well plates and incubated at 37 °C in a 5% CO_2_ incubator for 24 h. The media were then removed and the cells were treated with 100 µL of non-toxic concentration of mulberry leaf extract (312–1250 µg/mL), resveratrol (3.12–12.5 µg/mL), and oxyresveratrol (6.25–25 µg/mL) for 1 h, then LPS at 1 µg/mL (30 µL) was added to stimulate inflammation. After incubation for 24 h, the nitric oxide level in the cell culture medium was measured using Griess reaction assay [59]. Briefly, 100 µL of cell culture medium was mixed with 100 µL of Griess reagent (equal volumes of 1% (*w*/*v*) sulfanilamide in 5% (*v*/*v*) phosphoric acid and 0.1% (*w*/*v*) naphthyl ethylenediamine dihydrochloride), and incubated at room temperature for 15 min. Then the absorbance was measured at 540 nm by a microplate reader (DYNEX Technologies, Chantilly, VA, USA). The nitrite concentration was calculated using sodium nitrite as a standard within a range of 1 to 100 µM (y = 0.0144 x + 0.1501, R^2^ = 0.999).

*Treatment after stimulation with LPS*

The cells were plated at a density of 2 × 10^5^ cells/well in 96-well plates and incubated at 37 °C in a 5% CO_2_ incubator for 24 h. LPS stimulation was performed using LPS at 1 µg/mL (30 µL) for 10 min, then LPS was removed, followed by incubation with 100 µL of non-toxic concentration of mulberry leaf extract (312–1250 µg/mL), resveratrol (3.12–12.5 µg/mL), and oxyresveratrol (6.25–25 µg/mL) for 24 h. Nitric oxide levels in cell culture medium were measured using Griess reaction assay [59].

#### 4.5.4. RNA Extraction and cDNA Synthesis

The cells were plated at a density of 1.0 × 10^6^ cells/well in 24-well plates and stimulated with LPS at 1 µg/mL for 30 min prior to incubation with a non-toxic concentration of mulberry leaf extract (312–1250 µg/mL), resveratrol (3.12–12.5 µg/mL), and oxyresveratrol (6.25–25 µg/mL). Total RNA was isolated from the cells using Trizol^®^ reagent (Invitrogen, Carlsbad, CA, USA). The total RNA was converted into cDNA with a reverse transcription system containing 2 µL of 5× RT Master MIX (ReverTra Ace^®^ TOYOBO, Osaka, Japan), 2 µg total RNA, and 6 µL RNase-free water. The reaction was performed at 37 °C for 15 min, followed by incubation at 50 °C for 5 min and heating at 98 °C for 5 min, respectively.

#### 4.5.5. Real-Time Quantitative PCR Amplification

The cDNA was used for real-time qPCR amplification by SensiFAST™ SYBR^®^ No-ROX Kit (BIOLINE, London, UK) for analysis of *iNOS*, *COX-2*, *TNF-α*, and *IL-6*. The qPCR reaction system contained 10 µL SensiFAST SYBR^®^ (2×), 0.8 µL forward primer (10 µM), 0.8 µL reverse primer (10 µM), and 8.4 µL cDNA under the following reaction conditions: 95.0 °C for 2 min, followed by 40 cycles at 95.0 °C for 5 s and 65.0 °C for 30 s. The amplification results were expressed as the threshold cycle (Ct) value, which represented the number of cycles needed to generate a fluorescent signal greater than a predefined threshold. The expression levels of *iNOS*, *COX-2*, *TNF-α*, and *IL-6* were normalized using *β-actin* as an internal control. The primers for *iNOS*, *COX-2*, *TNF-α*, *IL-6*, and *β-actin* genes were used as shown in Table 3.

#### 4.5.6. Western Blotting Analysis

The cells were plated at a density of 1 × 10^6^ cells/well in 24-well plates and incubated at 37 °C in a 5% CO_2_ incubator for 24 h. LPS stimulation was performed at 1 µg/mL for 30 min prior to incubation with a non-toxic concentration of mulberry leaf extract (312–1250 µg/mL), resveratrol (3.12–12.5 µg/mL), and oxyresveratrol (6.25–25 µg/mL) for 12 h. Afterwards, cell suspension was centrifuged at 1200 rpm at 4 °C for 10 min. The cell pellet was washed with phosphate buffer saline (PBS) (pH of 7.4) twice by centrifugation at 1200 rpm for 5 min. After that, cell extraction was performed by adding 60 µL of RIPA lysis buffer (50 mM Tris-HCl, pH 8.0, 0.05 mM EDTA, 1% Triton X-100, 0.1% SDS, 150 mM NaCl, and distilled water) containing protease inhibitor cocktails with a ratio of 1:1000. The lysates were clarified by centrifuge at 3300 rpm at 4 °C for 15 min. The protein concentration was determined using BCA protein assay reagent (Merck, Billerica, MA, USA). The protein equivalents of samples (30 µg) were separated by sodium dodecyl sulfate polyacrylamide gel electrophoresis (15% SDS-PAGE) using Bio-Rad Mini-PROTEAN Tetra Handset (Hercules, CA, USA). SDS-PAGE gel electrophoresis was performed at 60 volts for 3 h 30 min to determine proteins and transferred to polyvinylidene fluoride (PVDF) membranes at 20 volts and 200 milliampere (mA) for 45 min using the semi-dry blotting method. The membranes were blocked with 5% skim milk for 1 h and incubated overnight at 4 °C with the primary antibodies, including iNOS (1:2000), COX-2 (1:1000), and β-actin (1:2000) by dilution in 5% skim milk. Then, membranes were incubated with horseradish peroxidase-conjugated secondary antibody (1:3000) for 1 h at room temperature. Antibody-specific proteins were visualized using enhanced chemiluminescence reagent (Immobilon Forte Western HRP Substrate, Merck, Billerica, MA, USA). Visualization of target proteins was detected through chemiluminescence detection (ImageQuant™ LAS 500, GE HealthCare, Chicago, IL, USA).

### 4.6. Statistical Analysis

Statistical analysis of data was performed using SPSS 17.0 statistical analysis software (IBM, Chicago, IL, USA). All quantitative data were verified in triplicate and presented as mean ± standard deviations. A one-way analysis of variance (ANOVA) was performed and the differences at *p* < 0.05 were considered statistically significant.

## 5. Conclusions

As a whole, white mulberry leaf extract appears to be a good source of phenolics and flavonoids that influence antioxidant activity, as determined by DPPH, ABTS, and FRAP assays. Although only oxyresveratrol was detected in this study, the crude mulberry leaf extract and its bioactive compounds demonstrated anti-inflammatory activity by suppressing nitric oxide generation in LPS-stimulated RAW 264.7 macrophage cells and substantially decreasing the protein expression of iNOS and COX-2 in a concentration-dependent manner. Additionally, pro-inflammatory cytokines, e.g., TNF-α and IL-6, were also downregulated. This study applied an in vitro approach to white mulberry leaf extract, as well as resveratrol and oxyresveratrol, regardless of its in vivo actions that may affect their potential anti-inflammatory activities. Moreover, the analysis of physiologically relevant reactive molecules, e.g., superoxide anions, hydrogen peroxide, and hydroxyl radicals, should be considered. As a result, animal models may help to investigate these issues and ultimately assess the benefits for human health.

## Figures and Tables

**Table 1:** Total phenolic and flavonoid content in white mulberry leaf extracts.

Cultivars	Total Phenolic Content (mg GAE/g Extract)	Total Flavonoid Content (mg QE/g Extract)
Sakon Nakhon	49.68 ± 1.41 ^a^	0.90 ± 0.01 ^b^
Buriram	27.27 ± 2.28 ^b^	1.46 ± 0.27 ^a^

Each value in the table is represented as mean ± SD (*n* = 3); ^a,b^ indicate significant difference (*p* < 0.05).

**Table 2:** Evaluation of antioxidant activities of white mulberry leaf extracts from DPPH, ABTS, and FRAP assays.

Cultivars	DPPH		ABTS		FRAP (mg FeSO_4_/g Extract)
	IC_50_ (mg/mL)	Antioxidant Activity (mg GAE/g Extract)	IC_50_ (mg/mL)	Antioxidant Activity (mg TEAC/g Extract)	
Sakon Nakhon	0.78 ± 0.08 ^b^	4.38 ± 0.48 ^a^	4.89 ± 1.18 ^b^	4.53 ± 1.11 ^a^	92.78 ± 1.40 ^a^
Buriram	1.67 ± 0.21 ^a^	2.33 ± 0.62 ^b^	15.08 ± 3.76 ^a^	1.47 ± 0.36 ^b^	69.30 ± 3.70 ^b^

Each value in the table is represented as mean ± SD (*n* = 3); ^a,b^ indicate significant difference (*p* < 0.05).

**Table 3:** The primers used for real-time PCR analysis.

Genes	Sense Primer Sequence 5′-3′	Antisense Primer Sequence 5′-3′	References
*iNOS*	TTCCAGAATCCCTGGACAAGC	TGGTCAAACTCTTGGGGTTCG	[60]
*COX-2*	AGAAGGAAATGGCTGCAGAA	GCTCGGCTTCCAGTATTGAG	[60]
*TNF-α*	AGCCCCCAGTCTGTATCCTTC	CATTCGAGGCTCCAGTGAATTCG	[60]
*IL-6*	GCTGGAGTCACAGAAGGAGTG	GCATAACGCACTAGGTTTGCC	[61]
*β-actin*	TGCTGTCCCTGTATGCCTCTG	GCTGTAGCCACGCTCGGTCA	[62]

## Data Availability

The data presented in this study are available on request from the corresponding author.

## Sample Availability

Samples of the mulberry leaf extracts are available from the authors.
